# The Dietary Polysaccharide Maltodextrin Promotes *Salmonella* Survival and Mucosal Colonization in Mice

**DOI:** 10.1371/journal.pone.0101789

**Published:** 2014-07-07

**Authors:** Kourtney P. Nickerson, Craig R. Homer, Sean P. Kessler, Laura J. Dixon, Amrita Kabi, Ilyssa O. Gordon, Erin E. Johnson, Carol A. de la Motte, Christine McDonald

**Affiliations:** 1 Department of Pathobiology, Lerner Research Institute, Cleveland Clinic, Cleveland, Ohio, United States of America; 2 Department of Molecular Medicine, Cleveland Clinic Lerner College of Medicine, Case Western Reserve University, Cleveland, Ohio, United States of America; 3 Department of Anatomic Pathology, Robert J. Tomsich Pathology & Laboratory Medicine Institute, Cleveland Clinic, Cleveland, Ohio, United States of America; 4 Department of Biology, John Carroll University, University Heights, Ohio, United States of America; University of Cologne, Germany

## Abstract

In the latter half of the 20^th^ century, societal and technological changes led to a shift in the composition of the American diet to include a greater proportion of processed, pre-packaged foods high in fat and carbohydrates, and low in dietary fiber (a “Western diet”). Over the same time period, there have been parallel increases in *Salmonella* gastroenteritis cases and a broad range of chronic inflammatory diseases associated with intestinal dysbiosis. Several polysaccharide food additives are linked to bacterially-driven intestinal inflammation and may contribute to the pathogenic effects of a Western diet. Therefore, we examined the effect of a ubiquitous polysaccharide food additive, maltodextrin (MDX), on clearance of the enteric pathogen *Salmonella* using both *in vitro* and *in vivo* infection models. When examined *in vitro*, murine bone marrow-derived macrophages exposed to MDX had altered vesicular trafficking, suppressed NAPDH oxidase expression, and reduced recruitment of NADPH oxidase to *Salmonella*-containing vesicles, which resulted in persistence of *Salmonella* in enlarged Rab7^+^ late endosomal vesicles. *In vivo*, mice consuming MDX-supplemented water had a breakdown of the anti-microbial mucous layer separating gut bacteria from the intestinal epithelium surface. Additionally, oral infection of these mice with *Salmonella* resulted in increased cecal bacterial loads and enrichment of lamina propria cells harboring large Rab7^+^ vesicles. These findings indicate that consumption of processed foods containing the polysaccharide MDX contributes to suppression of intestinal anti-microbial defense mechanisms and may be an environmental priming factor for the development of chronic inflammatory disease.

## Introduction

The composition of the American diet shifted dramatically in the latter half of the 20^th^ century to include a greater proportion of processed, pre-packaged foods high in fat and carbohydrates, with low dietary fiber (a “Western diet”). Over the same time period, a steady increase in nontyphoidal salmonellosis has been reported and *Salmonella spp*. are now the most common cause of foodborne bacterial disease outbreaks in the United States, with over one million cases reported in 2013 [1]. Concurrently, there have been increases in a broad range of chronic inflammatory diseases, such as inflammatory bowel disease, diabetes, asthma, and atherosclerosis [2]. These chronic inflammatory diseases are associated with intestinal microbial dysbiosis, which contributes to disease pathogenesis [3], [4]. Additionally, epidemiologic studies correlate Western diet consumption with an increased risk of death due to cardiovascular disease and cancer [5], [6]. Taken together, these findings suggest a relationship between consumption of a Western diet and multiple diseases.

A Western diet incorporates processed food constituents to promote longer shelf-life, improve texture, or enhance flavor, which may have detrimental effects on health. Consumption of a Western diet has been shown to not only alter the intestinal microbiota, but also influence cellular anti-microbial defense mechanisms. For example, case reports link outbreaks of necrotizing enterocolitis in preterm infants with consumption of a xanthan gum-derived food thickener [7], [8]. Additional studies have linked other thickening or emulsifying agents, such as carob-bean gum, modified starch, carboxymethyl cellulose, carrageenen, pectin, and cellulose to bacterially-driven intestinal diseases [9]–[15]. These findings suggest that food additives may alter anti-bacterial response mechanisms important in maintaining intestinal homeostasis.

*Salmonella* is a facultative intracellular anaerobe that causes both enteric, as well as systemic disease in humans. Both host and microbial factors which affect clearance of this pathogen are commonly investigated *in vivo* using mice pre-treated with streptomycin before oral inoculation with *Salmonella* [16] or by *in vitro* studies of infected macrophages or epithelial cell lines [17]. *Salmonella* has the ability to infect and replicate in both non-phagocytic and phagocytic cells through the injection of effector proteins by type III secretion systems. Once internalized, *Salmonella* traffics through the endosomal system to a specialized compartment, characterized by late endosomal and lysosomal membrane markers, but without luminal lysosomal hydrolases (the *Salmonella*-containing vesicle; SCV) [17]. The SCV is a protected niche for replication maintained by a second wave of bacterial effector proteins, which interfere with cellular defense mechanisms, such as a NADPH oxidase-induced respiratory burst [23]. Maintenance of this protected niche is important for both persistent infection and systemic spread of the bacterium [18].

Maltodextrin (MDX) is a polysaccharide commonly added to processed foods, cosmetics, and medications as a filler, thickener, texturizer, or coating agent. MDX is created through chemical and enzymatic processing of a variety of starches to produce chains of up to 20 glucose molecules linked by α(1–4) and α(1–6) glycosidic bonds [19]. It is generally recognized as safe (GRAS) by the Federal Drug Administration [20]; however, there are links between MDX and alterations in intestinal microbiota, as well as increased necrotizing enterocolitis in animal models [14], [21], [22]. To examine the contribution of this ubiquitous dietary additive to bacterially-driven disease, we investigated the effects of MDX on clearance of the enteric pathogen *Salmonella enterica* serovar Typhimurium in mice using both *in vivo* and *in vitro* infection models.

## Results

### MDX exposure impairs *Salmonella* clearance from intestinal epithelial cells and macrophages

**Figure 1:**
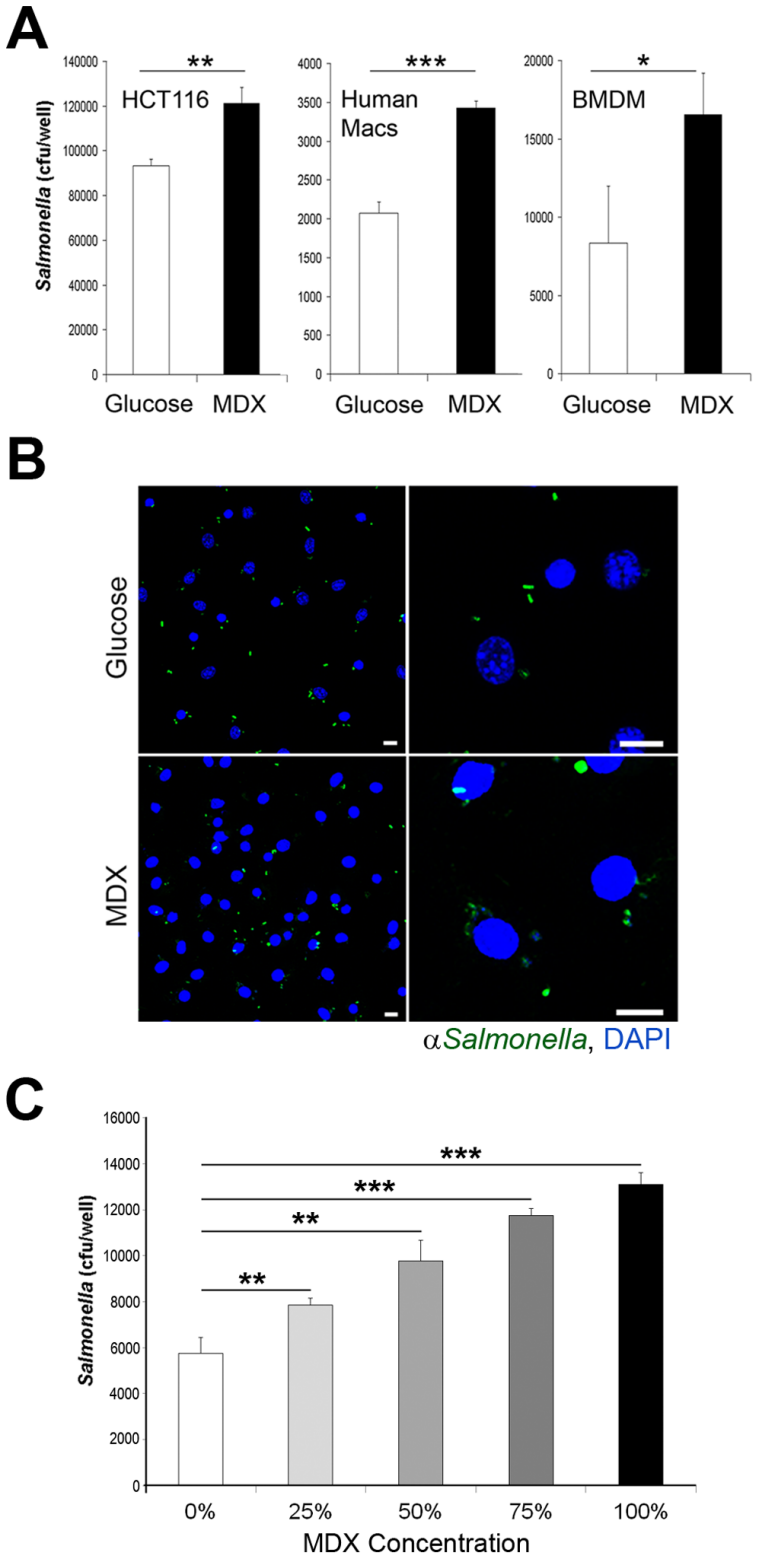
MDX exposure impairs *Salmonella* clearance in multiple cell types. (**A**) Recovery of intracellular *Salmonella* in gentamycin protection assays from the indicated cell types cultured in glucose or MDX supplemented media. (**B**) Confocal micrographs of infected BMDM stained for *Salmonella* (green). Nuclei stained with DAPI (blue). Scale bars: 10 µm. (**C**) Recovery of intracellular *Salmonella* in gentamycin protection assays from BMDM cultured in media reconstituted with a mixtures of MDX and glucose (i.e. 25% MDX/75% glucose for a total of 4.5 g/L). Data represented as mean±SD. *p<0.05, **p<0.01, ***p<0.001.

Consumption of a Western diet has been shown to not only alter the intestinal microbiota, but also influence cellular anti-microbial defense mechanisms [23]–[25]. To determine whether MDX affects bacterial clearance in cell types critical to intestinal defense, a variety of cell types were cultured in glucose-free media reconstituted with MDX or glucose for 24 hours and infected with *Salmonella*. Intracellular bacterial survival was determined by gentamycin protection assay 90 minutes post-infection. MDX exposure enhanced intracellular *Salmonella* survival in human intestinal epithelial cell lines, primary human monocyte-derived macrophages, and murine bone marrow-derived macrophages (BMDM) (Figure 1A & S1A). These results were confirmed by visualization of bacterial loads in BMDM by confocal microscopy (Figure 1B). The effects of MDX on *Salmonella* clearance were also dose-dependent, with impaired clearance observed in BMDM cultured in media reconstituted with a 25% MDX/75% glucose mix and further suppressed as the proportion of MDX increased in the media (Figure 1C). Furthermore, the observed suppression of *Salmonella* clearance after MDX exposure was not a result of glucose starvation, as cells cultured in glucose-free media had similar intracellular bacterial loads as glucose-supplemented cells (Figure S1A). These results indicate that MDX exposure promotes intracellular *Salmonella* persistence in multiple cell types involved in gut defense.

### *Salmonella* entry and trafficking to early endosomes are unaffected by MDX exposure

**Figure 2:**
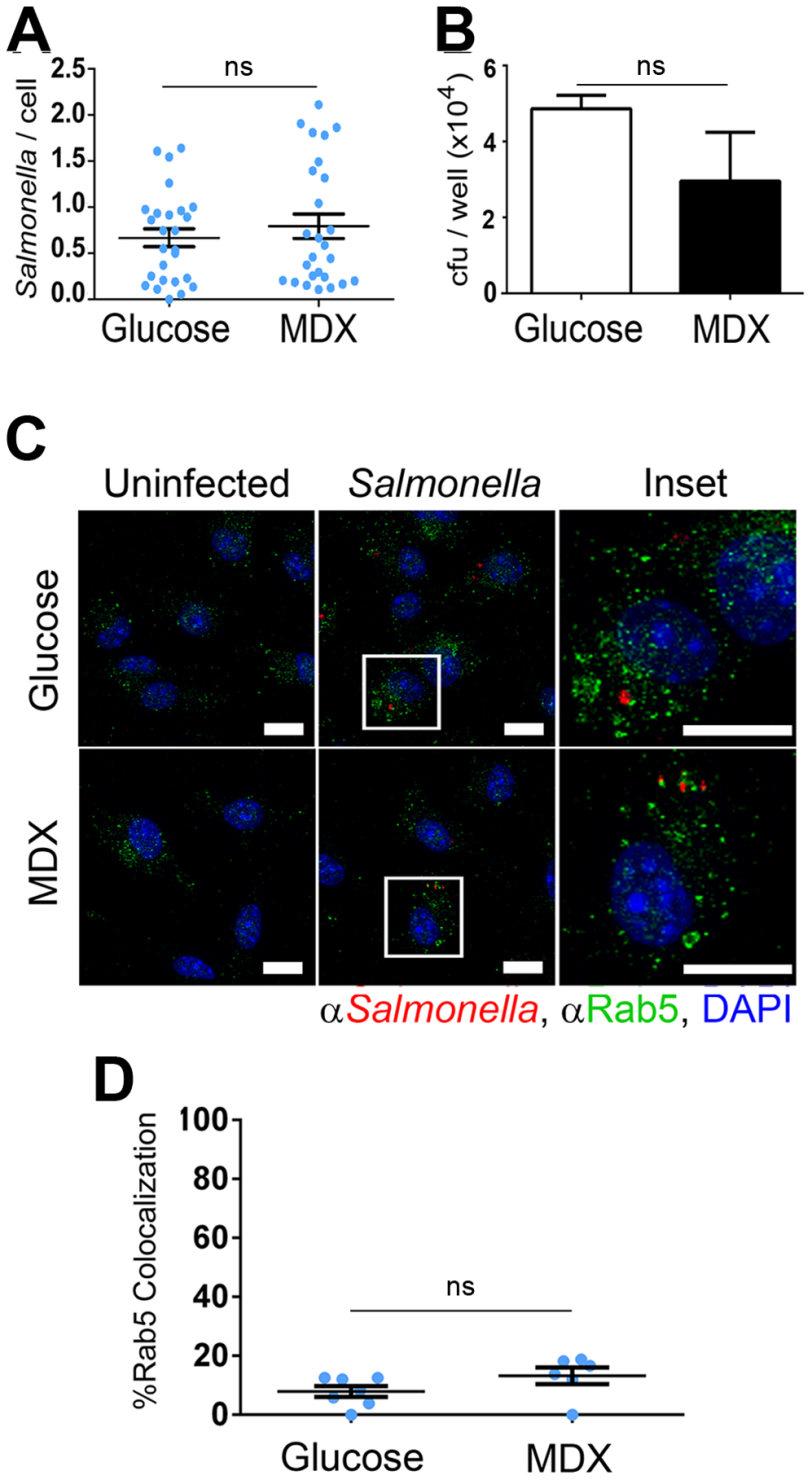
MDX does not affect *Salmonella* entry or trafficking to early endosomes. (**A**) Quantitation of the number of *Salmonella* per BMDM 30 minutes post-infection in confocal micrographs. Data represented as mean±SEM (**B**) Recovery of viable intracellular *Salmonella* from BMDM 30 minutes post-infection. Data represented as mean±SD (**C**) Confocal micrographs of BMDM 15 minutes post-infection stained for *Salmonella* (red) and Rab5 (green). Nuclei stained with DAPI (blue). Scale bars: 10 µm. (**D**) Quantitation of the number of co-localized *Salmonella* with Rab5^+^ vesicles per BMDM 15 minutes post-infection in confocal micrographs. Data represented as mean±SEM, ns = not statistically significant.

Increased intracellular bacterial loads in MDX exposed cells could be a result of defective bacterial clearance or enhanced bacterial entry. To discriminate between these two possibilities, the initial cellular entry of *Salmonella* was assessed by confocal microscopy and gentamycin protection assays in BMDM. No discernible differences in intracellular *Salmonella* numbers were found between BMDM cultured in glucose- or MDX-containing media by quantification of confocal micrographs or by recovery of viable intracellular bacteria 30 minutes post-infection (Figure 2A & 2B). Further analyses of confocal micrographs showed that *Salmonella* is immediately trafficked to a Rab5^+^ early endosome in both media conditions (Figure 2C & 2D). As early as 15 minutes post-infection, equivalent numbers of *Salmonella* co-localize with Rab5^+^ vesicles in both control and MDX exposed cells (Figure 2D). These findings indicate that MDX does not affect *Salmonella* entry or early trafficking events.

### MDX promotes a new replicative niche for *Salmonella*

*Salmonella* is a facultative intracellular pathogen that replicates within specialized, *Salmonella*-containing vesicles (SCV) [17]. These SCV acquire markers of late endosomes (Rab7) and selective lysosome-associated membrane proteins (LAMPs) without accumulation of lysosomal hydrolytic enzymes through the action of bacterial effector proteins [26]. The ability to form and maintain SCV has been correlated with both the pathogenicity of *Salmonella* strains, as well as host susceptibility to infection, indicating both bacterial and cellular factors contribute to this process [27].

**Figure 3:**
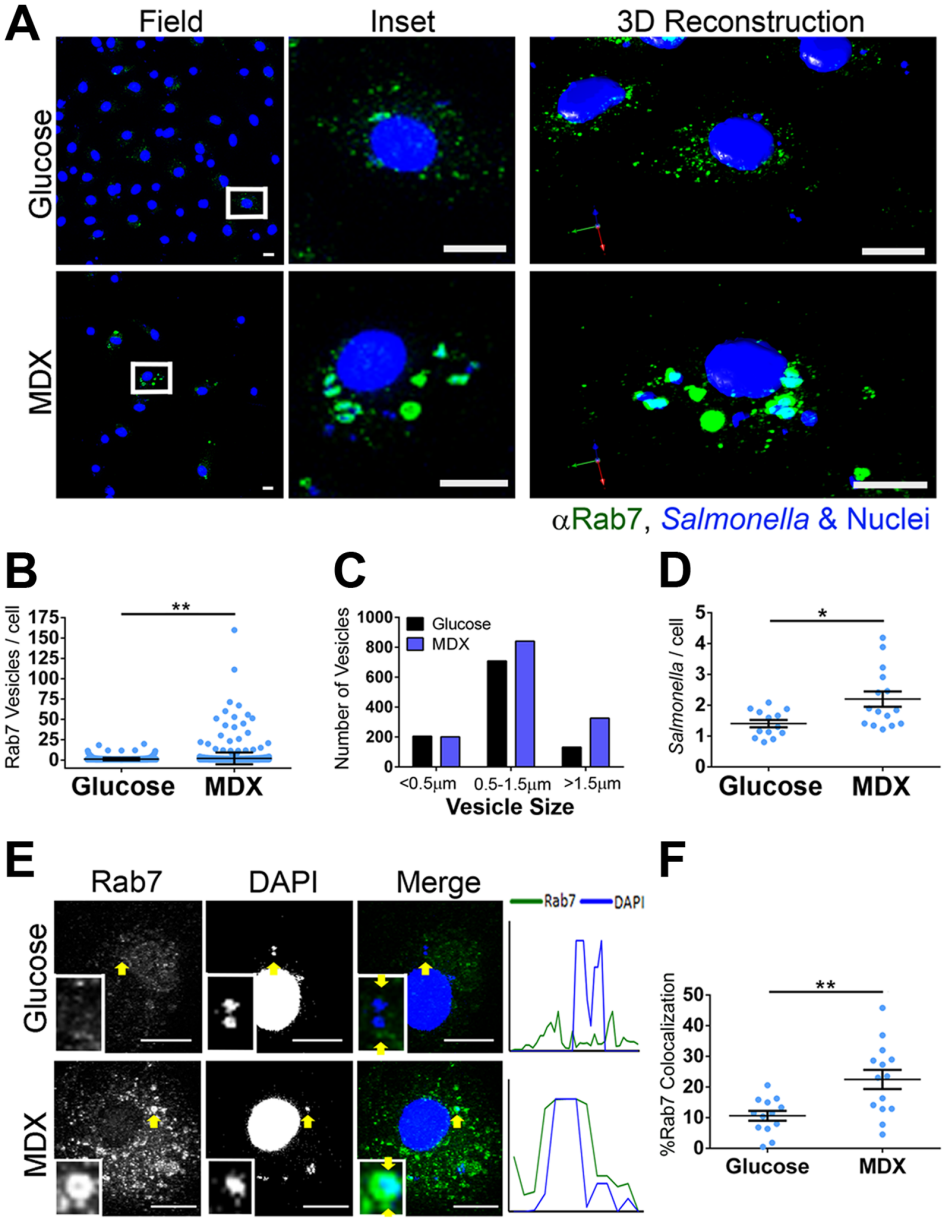
MDX promotes formation of enlarged Rab7^+^ *Salmonella*-containing vesicles. (**A**) Confocal micrographs of BMDM 90 minutes post-infection stained for Rab7 (green). *Salmonella* and nuclei stained with DAPI (blue). Scale bars: 10 µM (**B**) Quantitation of the number of Rab7^+^ vesicles per BMDM 90 minutes post-infection from confocal micrographs. Data represented as mean±SEM. (**C**) Quantitation of the size distribution of Rab7+ vesicles 90 minutes post-infection from confocal micrographs. Data represented as total number of vesicles scored from 103 cells scored. (**D**) Quantitation of the total number of *Salmonella* per BMDM 90 minutes post-infection from confocal micrographs. Data represented as mean±SEM. (**E**) Confocal micrographs of BMDM 90 minutes post-infection stained for Rab7 (green). *Salmonella* and nuclei stained with DAPI (blue). Histogram profiles were generated along a line connecting the arrows in the merged insets. (**F**) Quantitation of co-localized *Salmonella* with Rab7^+^ vesicles per BMDM 90 minutes post-infection by analysis of confocal micrographs. Scale bars: 10 µm. Data represented as mean±SEM. *p<0.05, **p<0.01.

**Figure 4:**
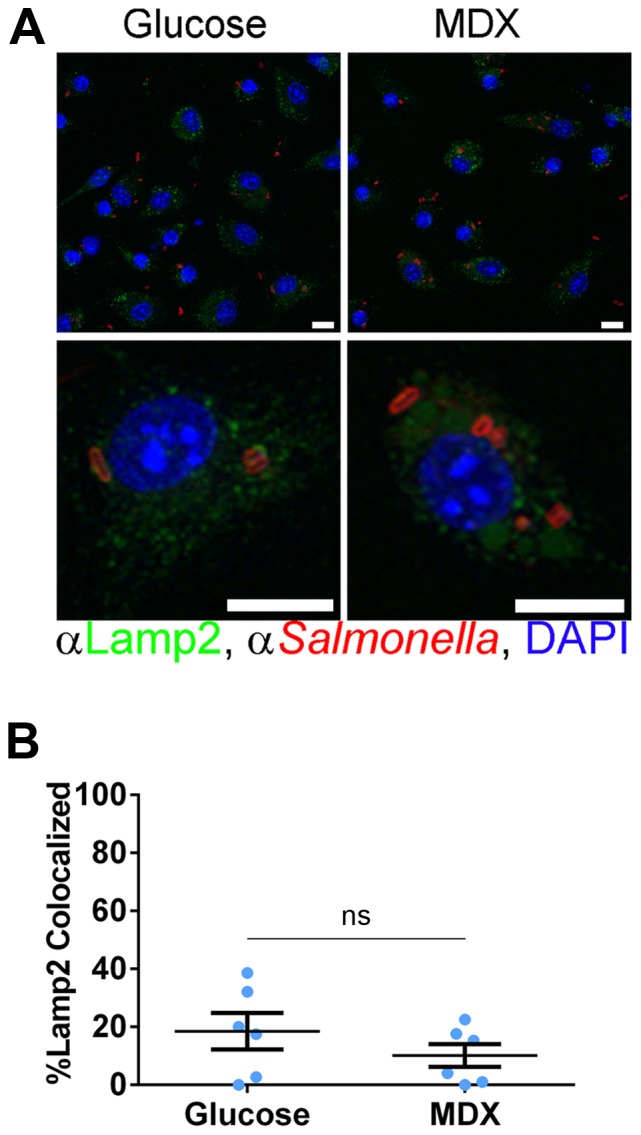
MDX does not block SCV maturation. (**A**) Confocal micrographs of BMDM 90 minutes post-infection stained for Lamp2 (green) and *Salmonella* (red). Nuclei stained with DAPI (blue). Scale bars: 10 µm. (**B**) Quantitation of the number of *Salmonella* co-localizing with Lamp2^+^ vesicles per BMDM 90 minutes post-infection from confocal micrographs. Data represented as mean±SEM, ns = not statistically significant.

The co-localization of *Salmonella* with Rab7^+^ vesicles was assessed in BMDM cultured in glucose- or MDX-supplemented media. At 90 minutes post-infection, a dramatic difference in the total number of Rab7^+^ vesicles was apparent (∼2 fold increase; Figure 3A & 3B). In addition to a greater intracellular *Salmonella* load, MDX exposed BMDM had more enlarged (>0.5 µm), Rab7^+^ vesicles as compared to glucose cultured cells (Figure 3C & 3D). The formation of these enlarged Rab7^+^ vesicles was not a response to cellular starvation, as they were not observed in *Salmonella* infected BMDM cultured in glucose-free media (Figure S1B). Quantitation of confocal micrographs demonstrated an enrichment of *Salmonella* within large, Rab7^+^ vesicles in MDX cultured cells (Figure 3E & 3F). Rab7 is essential for the recruitment and transfer of LAMPs to the SCV [26]. Surprisingly, although MDX enhanced Rab7 accumulation on SCVs, the total number of *Salmonella* co-localized with Lamp2^+^ vesicles was not different between glucose or MDX exposed cells (Figure 4A & 4B). These results suggest that MDX does not block SCV maturation, but promotes accumulation of *Salmonella* in enlarged Rab7^+^ vesicles that may function as a new replicative niche for these bacteria.

### MDX suppresses reactive oxygen species generation by reducing NAPDH oxidase expression and recruitment to SCVs

**Figure 5:**
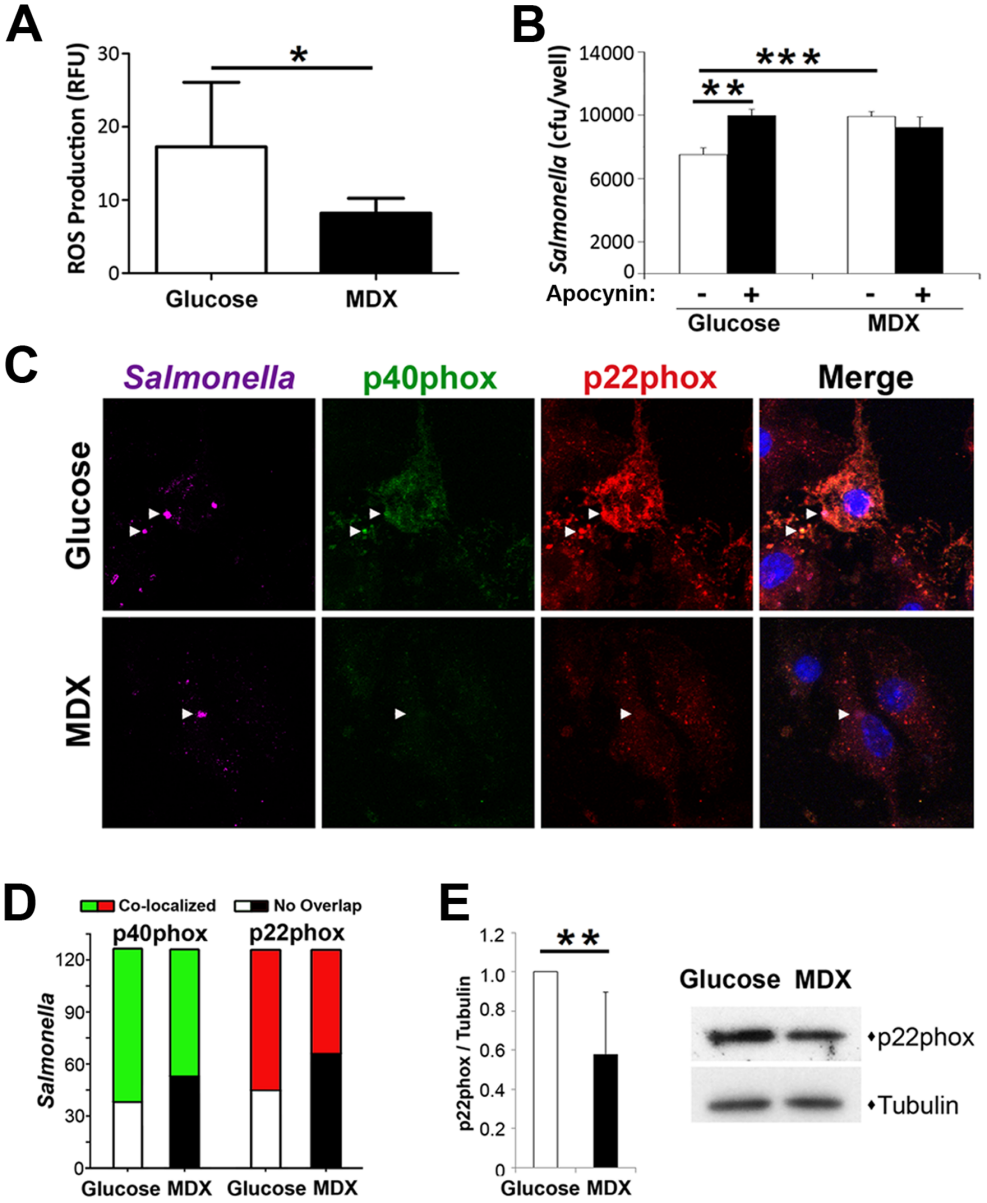
MDX suppresses ROS generation by reducing NAPDH oxidase expression and recruitment to SCVs. (**A**) ROS generation of BMDM 10 minutes post H_2_O_2_ stimulation. Data represented as mean±SEM. *p<0.05 (**B**) Recovery of viable intracellular *Salmonella* from BMDM pretreated with or without 1 µM apocynin by gentamycin protection assay 90 minutes post-infection. Data represented as mean±SD. ***p<0.001 (**C**) Confocal micrographs of infected BMDM 90 minutes post-infection stained for p40phox (green), p22phox (red), and *Salmonella* (purple; location indicated by arrowheads in all micrographs). Nuclei stained with DAPI (blue). Scale bars: 10 µm. (**D**) Quantitation of the co-localization of *Salmonella* with NADPH oxidase subunits in confocal micrographs of BMDM 90 minutes post-infection (total of 125 *Salmonella* scored per condition). (**E**) Quantification of p22phox expression levels in MDX-exposed BMDM relative to glucose cultured controls (n = 6). Immunoblot expression levels normalized to tubulin loading control. Data represented as mean±SD. **p<0.01. Representative immunoblot of p22phox expression levels in uninfected BMDM cultured in glucose or MDX supplemented media for 24 hours.

*Salmonella* target anti-microbial effectors, such as NADPH oxidase and inducible nitric oxide synthase (iNOS) to create a protective niche for replication [28]. Basal iNOS expression in BMDM was not altered by MDX exposure (data not shown); however, BMDM cultured in MDX-containing media had ∼50% reduction of reactive oxygen species (ROS) production in response to hydrogen peroxide, suggesting an impairment of NADPH oxidase function (Figure 5A). Complementing this result, chemical inhibition of NADPH oxidase with apocynin impaired *Salmonella* clearance in BMDM cultured in glucose-containing media, but did not affect clearance in MDX exposed BMDM (Figure 5B). These data suggest that MDX suppresses ROS generation by NAPDH oxidase.

NADPH oxidase is a multi-protein complex comprised of two membrane-associated components (gp91phox and p22phox), which recruit three cytosolic components (p47phox, p67phox, and p40phox) to intracellular vesicles to generate ROS [29]. To determine whether decreased ROS production in MDX exposed BMDM was due to altered recruitment of the NADPH oxidase complex to SCVs, the co-localization of *Salmonella* with p40phox or p22phox was assessed by confocal microscopy. Quantification of confocal micrographs demonstrated a decrease in the number of *Salmonella* co-staining with either p40phox or p22phox after exposure to MDX (Figure 5C & 5D). Additionally, BMDM cultured in MDX-containing media consistently had a lower staining intensity of both NADPH oxidase subunits, suggesting a decreased expression level. When protein levels of p22phox were assessed by immunoblot, MDX-exposed BMDMs expressed on average ∼40% less p22phox protein relative to cells cultured in glucose-containing media (Figure 5E). These results indicate that in addition to altering *Salmonella* trafficking, MDX exposure promotes *Salmonella* survival by decreasing both NADPH oxidase levels and localization of this complex to SCV.

### Dietary MDX consumption alters the intestinal anti-microbial barrier and enhances mucosal *Salmonella* colonization

**Figure 6:**
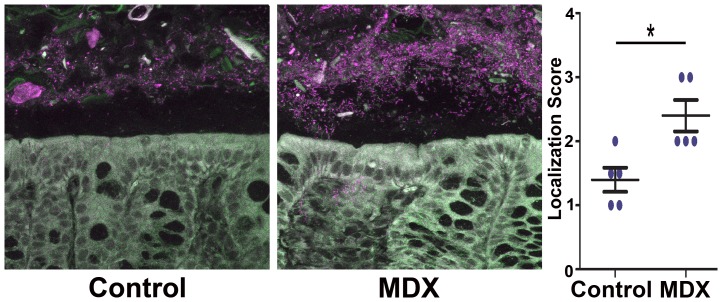
Dietary MDX consumption alters the intestinal anti-microbial barrier. Confocal micrographs of colonic sections from mice exposed to water (Control) or 5% MDX-supplemented water for 2 weeks visualizing the location of the commensal bacteria by FISH using a Eub338 probe (purple). Infiltration of bacteria into the anti-microbial barrier quantitated using an established scoring system (n = 5) [32]. Data represented as mean±SEM. *p<0.05.

The effect of MDX consumption on the clearance of an enteric pathogen *in vivo* was assessed using the streptomycin pre-treatment mouse model of *Salmonella* colitis [16]. The drinking water of 6 week old female C57BL/6 mice was supplemented with 5% MDX for 2 weeks. This concentration of MDX was selected based on the amount of MDX commonly used in infant formulas (55.5 g/L). Although daily water consumption was higher in the MDX-supplemented group (14.5+/−3.2 mL/cage vs. 25.7+/−4.1 mL/cage; p<0.00001), this did not translate into a difference in weight after 2 weeks (18.3+/−0.8 g vs. 18.8+/−2.4 g; p = 0.6). However, dramatic changes were observed in the localization of the intestinal commensal microbiota in uninfected mice (Figure 6). In the healthy intestine, the commensal microbiota is spatially separated from the intestinal epithelium by a layer of mucous enriched with anti-microbial factors [30], [31]. This anti-microbial zone is reduced or lost in many disease states, including inflammatory bowel disease, enteric infection, cancer, and cystic fibrosis [31]. In our mouse model, the location of commensal bacteria was visualized in colonic sections of streptomycin-treated mice by fluorescent *in situ* hybridization (FISH) using a universal eubacteria probe (Figure 6). Confocal micrographs visualized a striking decrease in the separation of bacteria from the epithelial layer in MDX-supplemented mice. The enhanced infiltration of bacteria into the anti-microbial zone in these mice was quantitatively confirmed using an established scoring system [32], suggesting that MDX consumption alters the intestinal anti-microbial barrier.

**Figure 7:**
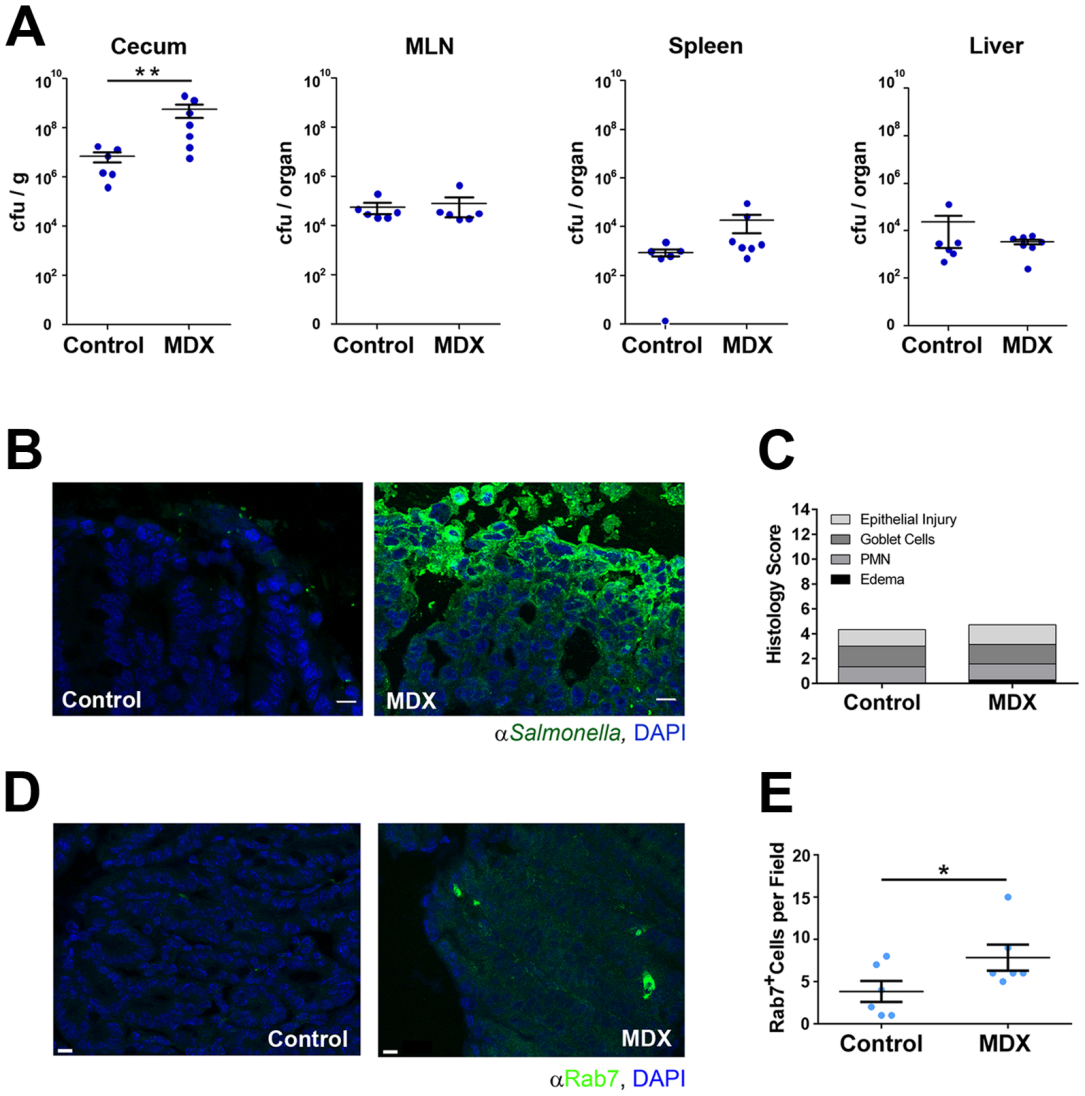
Dietary MDX consumption increases mucosal *Salmonella* colonization. (**A**) Quantitation of *Salmonella* recovered from cecum, MLN, spleen, and liver homogenates 48 hours post-infection of mice exposed to water (control) or 5% MDX-supplemented water for 2 weeks. Data represented as values from individual mice (dots; control n = 6; MDX n = 7) and mean±SEM. **p<0.01 (**B**) Confocal micrographs visualizing *Salmonella* colonization (green) in cecal tissue of control or MDX-supplemented mice. Nuclei stained with DAPI (blue). Scale bars: 100 µm. (**C**) Mean pathology scores of hematoxylin and eosin stained cecal tissue from infected mice. Pathology scores integrate analyses of epithelial integrity (Epithelial Injury), goblet cell hyperplasia (Goblet Cells), polymorphonuclear cell infiltration (PMN), and submucosal edema (Edema). (**D**) Confocal micrographs visualizing Rab7^+^ vesicles (green) in cecal tissue of control or MDX-supplemented mice. Nuclei stained with DAPI (blue). Scale bars: 100 µm. (**E**) Quantitation of the number of cecal lamina propria cells with Rab7^+^ vesicles. Six fields per group assessed, data represented as mean±SEM. *p<0.05.

Upon *Salmonella* infection, MDX-supplemented mice had enhanced cecal *Salmonella* colonization relative to control mice 48 hours post-infection, as demonstrated by a ∼2.5-log increase in viable bacteria recovered from cecal homogenates (Figure 7A). No differences were observed in colonization of systemic sites (MLN, spleen, or liver; Figure 7A). The enhanced *Salmonella* colonization of the cecum was confirmed by immunofluorescent staining of cecal tissue (Figure 7B). Increased cecal bacterial colonization did not translate into measurable differences in tissue pathology between treatment groups as assessed by histologic analysis of neutrophil accumulation, goblet cell depletion, edema and epithelial erosion using an established scoring system [16] (Figure 7C). However, increased numbers of lamina propria cells with large Rab7^+^ vesicles were observed in immunofluorescent confocal micrographs of cecal tissue sections from *Salmonella* infected mice consuming MDX-supplemented water relative to control mice (∼2 fold; Figure 7D & 7E). These findings indicate that the alterations of *Salmonella* trafficking and persistence induced by MDX *in vitro* also occur locally in the mucosa of infected mice consuming MDX.

## Discussion

Many dietary additives commonly incorporated into pre-packaged foods are registered as GRAS with the FDA, but they do not require formal evaluation by the FDA to validate safety. In the case of MDX, there is no limitation on its use in food other than a requirement for good manufacturing practices [20]. Increasingly, studies are linking several GRAS food additives to bacterially-driven intestinal diseases [7]–[15], [22]. Our findings demonstrate that MDX consumption may also contribute to bacterially-driven pathologies by reducing host anti-microbial defenses, resulting in a new niche for *Salmonella* survival in macrophages and the intestinal mucosa.

Macrophage function has been demonstrated to be suppressed by diet-induced obesity or high fat diet consumption through reduced iNOS induction and impaired ROS generation, respectively [19], [20]. Typically, *Salmonella* pathogenicity island-II (SPI-II) effector proteins are induced in a mature, acidified SCV and used by the bacteria to avoid clearance through preventing the localization of antimicrobial effectors, such as NADPH oxidase, to the SCV [17]. Our results demonstrate that MDX exposure reduces NADPH oxidase activity by both suppressing NAPDH oxidase subunit protein expression and localization to the SCV. It will be of interest to determine in future studies whether the formation of this novel protective niche for *Salmonella* in enlarged Rab7^+^ late endosomal vesicles is due primarily to permissive effects of MDX exposure on cells or whether MDX also enhances the pathogenicity of *Salmonella*.

Genetic variants in several NAPDH oxidase components have been associated with recurrent mycobacterial infections, chronic granulomatous disease, and Crohn's disease [24], [27], [28]. In our study, *in vitro* MDX exposure did not result in complete inhibition of NADPH oxidase activity, though it did result in a significant increase in viable *Salmonella*. Future studies in chronic infection models will shed light on whether this NADPH oxidase inhibition will result in granuloma formation or persistent infection. From our data, we would predict that chronic MDX consumption may increase susceptibility to recurrent bacterial infections or the development of granulomatous disease.

Our observation that mice fed MDX for a short time period have increased cecal bacterial loads upon oral *Salmonella* infection, but not enhanced systemic disease, suggests that MDX consumption alters intestinal homeostasis. Starches such as MDX are hydrolyzed by oral and ileal amylases, and are further degraded by epithelial cell membrane-bound maltases [33]. High MDX levels inhibit these brush border enzymes, further increasing luminal concentrations of MDX that can be used by intestinal microbes as a carbohydrate growth source [34]. Dysregulated bacterial overgrowth has been described in the small intestine, manifesting as debilitating abdominal cramping and gas production [4]. Small intestinal bacterial overgrowth (SIBO) is associated with several chronic diseases, including irritable bowel syndrome, inflammatory bowel disease, non-alcoholic steatohepatitis, scleroderma, diabetes, and diverticulitis [4]. Although it is not clear in all cases whether SIBO contributes to disease pathology or is a consequence of disease, there are several examples of diet promoting enhanced bacterial colonization, altered intestinal barrier function, and increased inflammation [10], [12]–[14], [23], [35]. Although we did not observe increased inflammation or intestinal damage at 48 h post infection in our MDX fed mice, the bacterial burden of these mice is similar to the enhanced cecal *Salmonella* colonization described in rats fed a “Western style diet” supplemented with dietary fructo-oligosaccharides or inulin [26], or the dysbiosis reported in mice fed a related Western diet (high fat, high sugar, and including MDX) infected with a Crohn's disease-associated pathobiont, adherent-invasive *Escherichia coli* LF82 [33]. These observations suggest that long-term MDX consumption may also contribute to the development of SIBO and the expansion of pathobionts.

The FDA does not currently track the consumption of GRAS food additives; therefore only limited information is available about the typical daily consumption of MDX. In a preliminary survey of grocery store shelf items, we found MDX (or modified starch) included in the ingredients list of approximately 50% of the items surveyed. For our *Salmonella* infection model, we provided mice with a similar amount of MDX typically found in infant formula; however actual consumption levels of humans may be much higher of this ubiquitous additive. Determination of a safe consumption threshold is of critical importance and may vary in the presence of additional disease risk factors (genetics, age, environmental insults, etc.). An example of this is the development of necrotizing enterocolitis in preterm infants after supplementation with a xanthan gum-derived food thickener commonly used safely in adults [7], [9]. Additionally, diets enriched with highly fermentable, but poorly absorbed, short chain carbohydrates and polyols (designated FODMAPs: Fermentable Oligo-, Di- and Mono-saccharides And Polyols) are now being proposed to promote Crohn's disease [20]. Conversely, exclusion diets and low complex carbohydrate diets, such as the specific carbohydrate diet (SCD), are showing promising results in relieving inflammatory bowel disease symptoms and improving disease activity scores [36]. We propose that these restrictive diets may be successful in part due to the exclusion of MDX or other additives from the diet and is a factor that should be examined more directly in future dietary intervention studies.

In summary, our findings demonstrate that MDX exposure promotes the formation of a novel protective niche for *Salmonella* through dampening host anti-microbial responses to enhance intracellular survival and mucosal colonization. These results suggest that consumption of processed foods containing the polysaccharide MDX may contribute to a greater risk for enteric infection may be an environmental priming factor for the development of chronic inflammatory diseases, such as inflammatory bowel disease [37].

## Materials and Methods

### Ethics Statement

All animal studies were approved by the Cleveland Clinic Institutional Animal Care and Use Committee (IACUC protocol#2013-0955) and performed in compliance with the US Department of Health and Human Services Guide for the Care and Use of Laboratory Animals. All primary human material used in this study was obtained from healthy donors by the Cleveland Clinic Clinical and Translational Sciences Collaborative using a protocol approved by the Cleveland Clinic Institutional Review Board (IRB#08-957). Written informed consent was obtained from all donors prior to participation.

### Cells and cell lines

All media and media supplements were obtained from Life Technologies (Grand Island, New York), except where indicated. The HCT116 cell line (gift of Gabriel Nuñez, University of Michigan, Ann Arbor, Michigan) was maintained in Dulbecco's Modified Eagle Medium (DMEM) with high glucose supplemented with 10% fetal bovine serum (FBS). The HT29 cell line was maintained in Roswell Park Memorial Institute Medium (RPMI) supplemented with 10% FBS. Primary human peripheral blood monocytes were isolated from healthy donors by counterflow centrifugal elutriation by the Cleveland Clinic Clinical and Translational Sciences Collaborative using a protocol approved by the Cleveland Clinic Institutional Review Board and differentiated into macrophages by culturing in RPMI supplemented with 10% FBS and 50 ng/mL macrophage colony stimulating factor for 7 days. Murine bone marrow-derived macrophages (BMDM) were isolated as previously described [38]. Briefly, bone marrow was flushed from femurs and tibias of C57BL/6J (Jackson Laboratories, Bar Harbor, Maine) or AKR mice (gift of Fabio Cominelli, Case Western Reserve University, Cleveland, Ohio; used in Figure S1A analyses only) and plated on Petri dishes in DMEM supplemented with 10% FBS, 30% L929 conditioned media, non-essential amino acids and penicillin/streptomycin. After 5 days, BMDM were replated for analysis in experimental media for 24 h. Experimental media consisted of DMEM without glucose supplemented with 10% FBS and glucose (4.5 g/L; Sigma, Saint Louis, Missouri), MDX (4.5 g/L; Spectrum Chemicals, New Brunswick, New Jersey), or mixtures of MDX and glucose (i.e. 25%MDX = 1.125 g/L MDX, 3.375 g/mL glucose for a total of 4.5 g/L) and was made fresh every 2 weeks.

### *In vitro* Salmonella infections

Cells were infected in triplicate with log phase *Salmonella enterica* serovar *Typhimurium* strain SL1344 at an MOI of 10 for 30 minutes followed by two washes in PBS and replacement of media containing 50 µg/mL gentamycin (Sigma). For experiments testing the effect of NADPH oxidase inhibition, cells were pretreated with 1 µM apocynin (Sigma) for 1 hour 20 minutes prior to infection. Cells were harvested in 0.1% Triton X-100/PBS 1 hour post-gentamycin addition and serial dilutions in LB broth plated on LB agar to determine viable intracellular *Salmonella* CFU.

### Immunofluorescent staining and confocal microscopy

BMDM were plated on glass coverslips in experimental media 24 hours prior to infection. At harvest, cells were fixed in 4% paraformaldehyde/PBS (Electron Microscopy Services, Hatfield, Pennsylvania), followed by permeabilization in 0.4% Triton X-100/PBS. Cells were subsequently blocked in 0.2% FBS/0.4% Triton X-100/PBS followed by addition of antibodies in 0.2% FBS/PBS. Antibodies used are as follows: polyclonal rabbit anti-Rab5 (#21435; Cell Signaling Technologies, Danvers, Maine), polyclonal rabbit anti-Rab7 (#20942; Cell Signaling Technologies), monoclonal mouse anti-Lamp2 (G12A7; Abcam, Cambridge, Massachusetts), monoclonal mouse anti-*Salmonella* (AbD Serotech, Raleigh, North Carolina), polyclonal rabbit anti-*Salmonella* (PA1-7244; Thermo Scientific, Waltham, Massachusetts), rabbit anti-p22phox (sc20781; Santa Cruz Biotechnology, Santa Cruz, California), goat anti-p40phox(sc18252; Santa Cruz Biotechnology), goat anti-rabbit-Alexa488, goat anti-rabbit-Alexa568, goat anti-mouse-Alexa488, goat anti-mouse-Alexa568, goat anti-rat-Alexa488, goat anti-mouse-Alexa633, goat anti-rat-Alexa633 (Life Technologies). After staining, coverslips were mounted on glass slide with Vectashield containing DAPI (Vector Laboratories, Burlingame, California) and sealed with nailpolish. Imaging was conducted using a Leica TCS-SP spectral laser scanning confocal microscope equipped with a Q-Imaging Retiga EXi cooled CCD camera and Image ProPlus Capture and Analysis software (Media Cybernetics, Rockville, Maryland). Image z-stacks were collected every 0.49 µm spanning the full thickness of cells and exported for image quantitation using Image ProPlus Capture and Analysis software (Media Cybernetics). Vesicle size, number and co-localization of staining were quantitated in an automated fashion using a customized visual basic Image-Pro Plus macro. Co-localization was verified by manual scoring of image stacks. A minimum of 2 fields were analyzed for each experimental condition and experiments were repeated at least three times. 3D reconstructions were prepared using Volocity software. Image stacks were imported and visualized using 3D projections and uniform percent-black and percent-brightness for all images analyzed and presented. Co-localization histogram traces were generated using ImageJ (National Institutes of Health, Bethesda, Maryland). For the images presented in Figure S1B, slides were visualized using a Leica DM200 fluorescence microscope and images acquired with a Retiga 2000R camera and QCapture Pro5.1 software (QImaging).

### Reactive oxygen species measurement

BMDM (2.5×10^5^ cells/mL) were plated in a 96 well plate 24 hours prior to analysis. Cells were washed in Hanks' Balanced Salt Solution and incubated with 10 mM Carboxy-H_2_DCFDA (C6827; Life Technologies) for 30 minutes in the dark. After washing, cells were stimulated with 100 mM H_2_O_2_ and fluorescence measured using a Spectromax plate reader recording excitation/emission 495/529 nm every 22 seconds at 37°C. Assay performed in triplicate in 4 independent experiments.

### Immunoblot analysis of NADPH oxidase expression

BMDMs (10^6^ cells/mL) were plated at into media containing glucose or MDX for 24 hours. Cells were lysed in Ginger buffer (310 mm Tris, pH 6.8, 25% glycerol, 5% SDS, 715 mm β-mercaptoethanol, 125 mg/ml bromphenol blue) on ice. The resulting lysate was separated by SDS-PAGE, and proteins were transferred to a PVDF membrane. Membranes were blocked in 5% milk, Tris-buffered saline, 0.1% Tween 20, and then probed with anti-p22phox (sc20781; Santa Cruz Biotechnology) or anti-tubulin (T9026; Sigma) overnight at 4°C. Blots were developed by enhanced chemiluminescence (Millipore, Billerica, Massachusetts). Protein levels were determined by densitometric analysis of scanned immunoblots (n = 6) using ImageJ.

### Salmonella infection *in vivo*

The experimental protocol was approved by the Cleveland Clinic Institutional Animal Care and Use Committee and performed in compliance with the US Department of Health and Human Services Guide for the Care and Use of Laboratory Animals. Female 6 week old C57BL/6J mice from Jackson Laboratories (stock number 000664) were fed Harlan Teklad Global Irradiated Rodent Diet 2918 and water supplemented with or without 5% (w/v) MDX (DE 4–7; Spectrum Chemicals) for 2 weeks prior to infection. Water consumption was monitored daily and animal weight measured every other day. Infection studies were performed as described in Barthel et al., 2003 [16]. One day prior to infection, food and water were withdrawn for 4 hours, and mice were orally gavaged with 75 µL streptomycin (20 mg) in water. Twenty hours post-streptomycin treatment, food and water were withdrawn for 4 hours and mice infected with 10^8^ cfu *Salmonella enterica* serovar Typhimurium strain SL1344 (100 µL suspension of log-phase cultures in PBS) by gavage. Food and water were returned 2 hours after infection and mice monitored for 48 hours. Mice were sacrificed by CO_2_ asphyxiation and blood collected by cardiac puncture into microtainer EDTA tubes. Mesenteric lymph nodes (MLN), spleen and liver were harvested aseptically, weighed and homogenized in cold, sterile PBS for bacterial enumeration. Cecal content was removed and tissue was divided in half, weighed and either fixed in Histochoice (AMRESCO, Solon, Ohio) or homogenized in cold, sterile PBS. Tissue *Salmonella* loads were quantified by plating serial dilutions of tissue homogenate on MacConkey agar plates supplemented with streptomycin (50 µg/mL). Hematoxylin and eosin-stained cecal sections (5 µm thick) were evaluated for inflammation and pathology by a gastrointestinal pathologist blinded to treatment group using the scale detailed in Barthel et al., 2003 [16].

### Fluorescent *in situ* hybridization

Colonic tissue was fixed in methanol-based Carnoy's fixative (60% absolute methanol, 30% glacial acetic acid, 10% chloroform), embedded in paraffin blocks, and 5 µm sections mounted on glass slides. Slides were deparaffinized and hybridized with 500 ng Eub338-Alexa633 probe (or buffer-only controls) in 20 mM Tris-HCL, 0.01% SDS, 0.9 M NaCl at 46°C overnight [39]. Slides were then rinsed twice with water, incubated 5 minutes in 20 mM Tris-HCL, 0.9 M NaCl at 46°C, rinsed twice with water, then dried 10 minutes at 46°C and coverslips applied with Vectashield containing DAPI (Vector Labs). Sections were imaged as described for immunofluorescent confocal microscopy. Bacterial infiltration scores were assessed on a 4-point scale (1 =  preserved mucus layer and no bacterial contact with the epithelium, 2 =  bacterial penetration into mucus layer without epithelial contact, 3 =  some bacteria in contact with epithelium, and 4 =  extensive epithelial contact with the epithelium) as previously described Korn et al., 2014 [32].

### Statistical analysis

Statistical analyses were performed using Prism software (Graph Pad) and p values ≤0.05 were considered significant. *In vitro* experiments were performed in triplicate in a minimum of three independent experiments and analyzed by either ANOVA or two-tailed, unpaired t-test to determine significance. *In vivo* experiments were conducted with a total of 6 control mice and 7 experimental group mice and analyzed using a two-tailed Mann-Whitney test to account for unequal variance. Data is presented as averages with standard error unless noted in the figure legend.

## Supporting Information

Figure S1 **Glucose starvation does not affect Salmonella clearance or induce formation of enlarged Rab7^+^ vesicles.** (**A**) Recovery of intracellular Salmonella in gentamycin protection assays from the indicated cell types cultured in unsupplemented (No Sugar), glucose, or MDX supplemented media. Data represented as mean±SD. *p<0.05, **p<0.01. (**B**) Immunofluorescent micrographs of BMDM 90 minutes post-infection stained for Rab7 (green). Salmonella and nuclei stained with DAPI (blue). Scale bars: 10 µM.
(TIF)
