# Supplementary figures and images for: The Dietary Polysaccharide Maltodextrin Promotes Salmonella Survival and Mucosal Colonization in Mice
Source: PLoS One. 2014 Jul 7;9(7):e101789. doi: 10.1371/journal.pone.0101789 (PMC4084946; doi:10.1371/journal.pone.0101789)

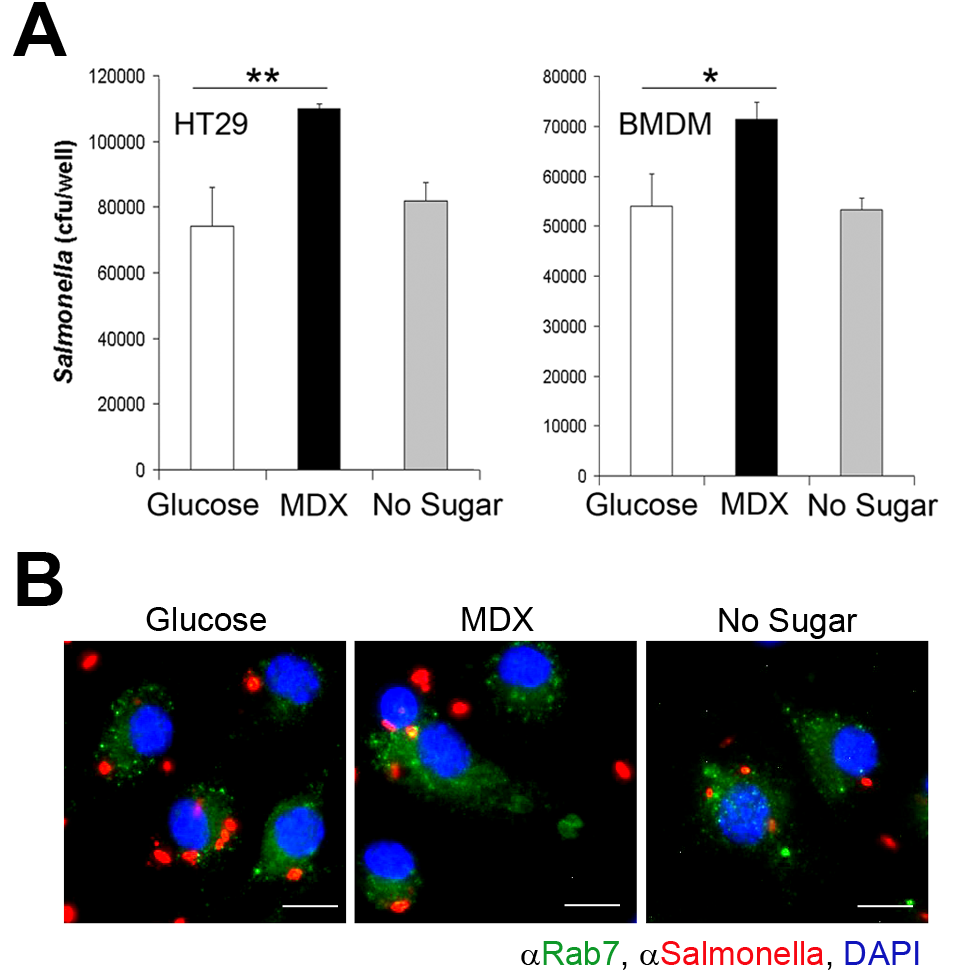

Supplement: Figure S1 — Glucose starvation does not affect Salmonella clearance or induce formation of enlarged Rab7+ vesicles. (A) Recovery of intracellular Salmonella in gentamycin protection assays from the indicated cell types cultured in unsupplemented (No Sugar), glucose, or MDX supplemented media. Data represented as mean±SD. *p<0.05, **p<0.01. (B) Immunofluorescent micrographs of BMDM 90 minutes post-infection stained for Rab7 (green). Salmonella and nuclei stained with DAPI (blue). Scale bars: 10 µM. (TIF) [file pone.0101789.s001.tif]
